# Epigenetics and endoplasmic reticulum in podocytopathy during diabetic nephropathy progression

**DOI:** 10.3389/fimmu.2022.1090989

**Published:** 2022-12-22

**Authors:** Xiaokang Wang, Jingqian Zhao, Yuanqing Li, Jiaoyu Rao, Gengrui Xu

**Affiliations:** ^1^ Department of Pharmacy, Shenzhen Longhua District Central Hospital, The Affiliated Central Hospital of Shenzhen Longhua District, Guangdong Medical University, Shenzhen, China; ^2^ Department of Pharmacy, Shenzhen Hospital, Southern Medical University, Shenzhen, China

**Keywords:** epigenetics, endoplasmic reticulum, podocyte, diabetic nephropathy, inflammation signaling

## Abstract

Proteinuria or nephrotic syndrome are symptoms of podocytopathies, kidney diseases caused by direct or indirect podocyte damage. Human health worldwide is threatened by diabetic nephropathy (DN), the leading cause of end-stage renal disease (ESRD) in the world. DN development and progression are largely dependent on inflammation. The effects of podocyte damage on metabolic disease and inflammatory disorders have been documented. Epigenetic and endoplasmic reticulum (ER) stress are also evident in DN. Targeting inflammation pathway and ER stress in podocytes may be a prospective therapy to prevent the progression of DN. Here, we review the mechanism of epigenetics and ER stress on podocyte inflammation and apoptosis, and discuss the potential amelioration of podocytopathies by regulating epigenetics and ER stress as well as by targeting inflammatory signaling, which provides a theoretical basis for drug development to ameliorate DN.

## Introduction

Diabetes mellitus (DM) is characterized by systemic hyperglycemia and can lead to a wide range of complications. It has seriously threatened the quality of life of millions worldwide. Although intensive measures have been taken to control blood glucose, the number of people with DM worldwide was half a billion in 2021 (1). Around 30–40% of type 1 or 2 DM patients are, unfortunately, prognosed with diabetic nephropathy (DN) and eventually develop end-stage renal disease (ESRD) (2, 3), imposing an enormous economic burden on individuals, families, and societies. Therefore, novel treatments for DN are urgently needed.

The roles of various pathways in the pathogenesis of DN have been established. Activation of the renin-angiotensin-aldosterone system (RAAS) (4), reactive oxygen species (ROS) (5) and inflammation (6) are related to the onset and development of DN (7). Although treatment with angiotensin-converting enzyme inhibitors (ACEI) or angiotensin receptor blockers (ARB) slows the progression of diabetic nephropathy to ESRD, the risk of ESRD remains high and is associated with residual proteinuria (8), suggesting that better strategies are needed to prevent DN. There has been a growing consensus that inflammation pathways are important in DN progression. New therapeutic strategies could be developed by identifying novel inflammatory factors (9). In patients with diabetic kidney disease (DKD), glomerular macrophage accumulation correlates strongly with progression of kidney impairment (10). During infiltration, macrophages release cytokines such as TNF, IL-1, and IFN-γ, accelerating the progression of DKD (11). By altering the viability of podocytes, macrophages contribute directly to DKD in bone-marrow chimeric mice (12). Genetic deficiency or pharmacological blockade of C-C chemokine receptor type 2 impairs macrophage recruitment to DKD in mouse models, reducing albuminuria and urea nitrogen levels as well as improving histological parameters. Treatment with the CCR2 inhibitor CCX140-B for 52 weeks significantly reduced urinary albumin excretion in T2DM patients with proteinuria (13). Angiotensinogen and cytokines are implicated in DKD—NF-κB initiates inflammation and stimulates the transcription of genes encoding cytokines and adhesion molecules (14). T2DM patients with NF-κB activation in muscle are likely to develop peripheral-tissue insulin resistance (15). DKD patients with bardoxolone have shown renoprotective effects with inhibitors of NF-κB (16). Therefore, research on small molecular targets of inflammatory signaling pathways in DN is needed.

Epigenetic modifications play a role in DKD, by affecting gene expression in response to environmental factors (17, 18). Epigenetic modifications enable mitotically and/or meiotically heritable changes in gene function without altering the underlying DNA sequence (19). Most disease-associated loci and single-nucleotide polymorphisms (SNPs) are found in non-coding regions of the genome, including regulatory regions such as enhancers and promoters, as well as in non-coding RNAs (20), which can affect gene expression by altering transcription factor binding and chromatin accessibility.

Podocytopathies is a key factor in DN development. And podocytopathies can lead to destruction of the glomerular basement membrane (GBM), inducing proteinuria (21). Mice with podocyte-specific conditional disruption of slit molecule genes suffer severe proteinuria and sclerosis (22). Proteinuria is closely associated with foot process effacement in minimal change disease (23). Moreover, albuminuria is diagnostic of early DN. Although podocyte injury is associated with the progression of DN (24), the underlying mechanisms are unclear. Interestingly, ER stress accelerates DN progression by injuring podocytes, endothelial cells, and mesangial cells (25, 26). Hyperglycemia and proteinuria (27) reportedly induce ER stress in DN. Persistent ER stress could affect ER function and induce a maladaptive unfolded protein response (UPR), activating apoptotic signaling pathways and causing podocyte apoptosis (28).

Taken together, the interaction of epigenetic modifications and ER stress with inflammation and apoptosis in podocytopathies is unclear. Studies on the epigenetic mechanism of DN (17) have attenuated ER stress by targeting long non-coding RNA (lncRNA) and/or microRNA. And epigenetics may regulate diabetes-related diseases under inflammatory conditions. A review of recent research on epigenetic mechanisms and endoplasmic reticulum stress in inflammation or apoptosis in podocytopathy presented here. Looking forward to provide new drug treatment strategies for podocytopathy during DN progression.

## Podocytopathies and DN

The glomerular filtration barrier is maintained by podocytes, highly specialized epithelial cells that contain critical molecules required for selective permeability (29). Loss of large numbers of podocytes leads to proteinuria, mesangial expansion, and glomerular sclerosis. Diabetic patients’ podocytes are susceptible to injury due to apoptosis, which is the most common mode of podocyte loss due to glucose-induced oxidative stress and advanced glycation end products (28, 30). However, little is known of the underlying mechanisms (31).

**Figure 1:**
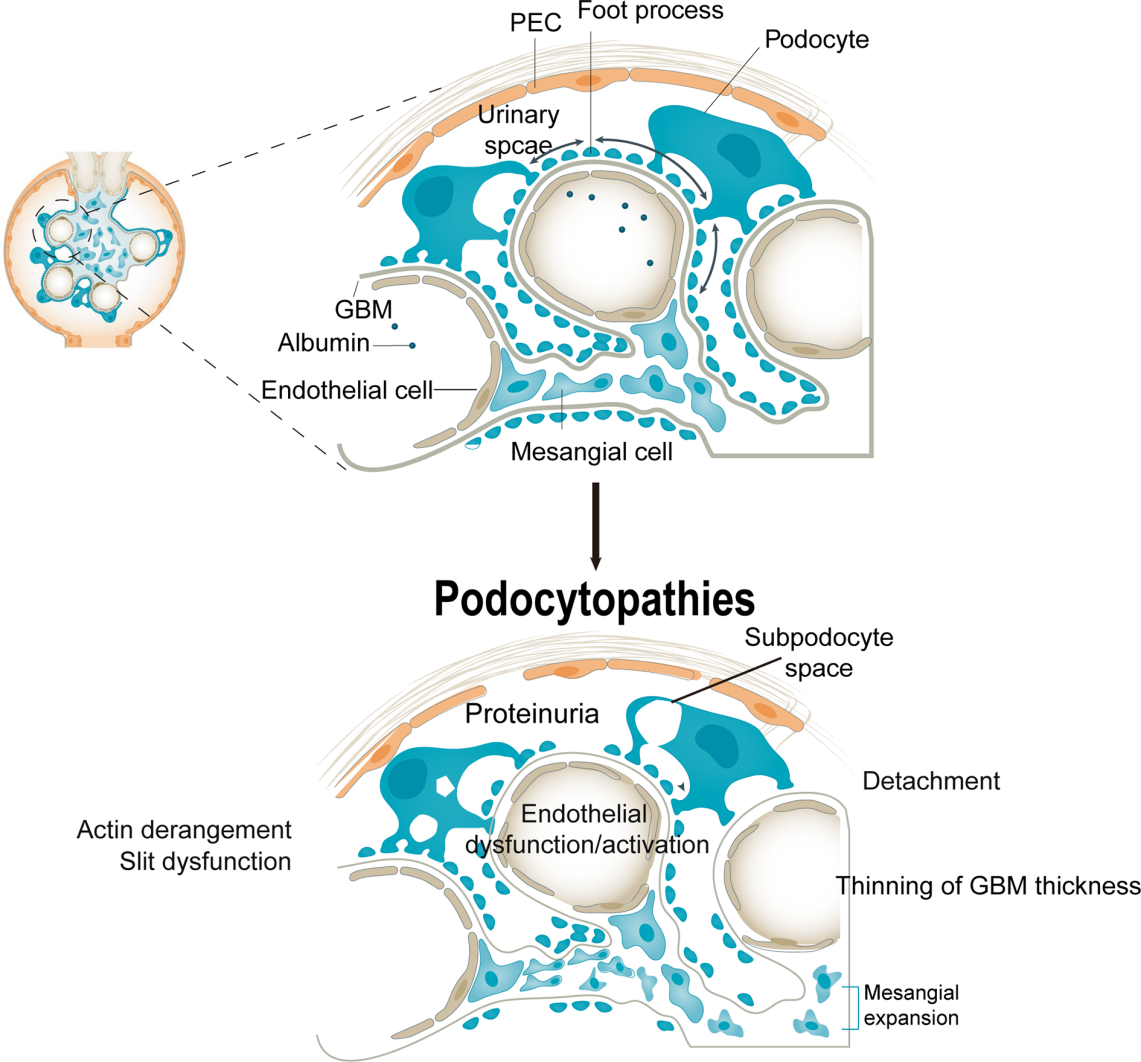
Consequences of podocytopathies. Podocyte injury causes cellular and structural responses in the glomerulus. An injured podocyte has cytoplasmic blebs, protein droplets, and an expanded subpodocyte space. Foot process effacement is associated with actin derangement, which induces dysfunction of slit molecules. The injured podocyte detaches from the glomerular basement membrane, which is associated with abnormalities of adhesion molecules, coagulation cascade, chemokine receptor expression, and lipid peroxidation. These changes may depend on podocyte detachment but also on aberrant factors from injured podocytes. PECs, parietal epithelial cells; VEGF, vascular endothelial growth factor.

Podocyte injury is characterized by hypertrophy, epithelial mesenchymal transition (32), detachment, and apoptosis (33). Glomerular hyperfiltration is accompanied by podocyte hypertrophy and abnormal podocyte function (34). Moreover, hyperglycemia could decrease the attachment of podocytes to the GBM—integrin α3β1 is an important receptor for podocyte–GBM attachment (35). The levels of podocyte cytoskeleton proteins, such as synaptopodin, podocin, and nephrin, were significantly reduced in diabetic kidneys (36). That is, a disordered podocyte cytoskeleton hampers adhesion, increasing GBM permeability and inducing proteinuria. Therefore, injury and depletion of podocytes is a crucial pathologic mechanism of albuminuria in DN. Proteinuria is also associated with a decrease in podocyte density and number in type 1 and type 2 diabetes. Indeed, podocytes in urine are an earlier marker of DN than proteinuria (37) (**Figure 1**).

Podocyte apoptosis contributes to filtration dysfunction in DN. As highly differentiated cells, podocytes have limited self-renewal *via* mitosis under adverse conditions, rendering them vulnerable to damage by stress (28, 38). Therefore, podocytes are dependent on clearance mechanisms such as the adaptive UPR and autophagy to restore intracellular hemostasis (38). Podocyte autophagy deficiency triggers proteinuria and glomerulosclerosis, and maintaining a certain level of autophagy ensures normal physiological function (39). In aldosterone-induced ER stress and podocyte injury, inhibition and induction of ER stress suppress and enhance, respectively, autophagy (40). Hence, restoration of podocyte structure and function after injury is important.

## Epigenetics in podocytopathies during DN progression

### Histone modifications

Histone modifications (acetylation, methylation, phosphorylation, ubiquitination, and carbonylation) are important in epigenetics; histone acetylation and methylation predominate. Inhibitors of histone deacetylases (HDACs) (such as valproic acid) and SAHA (such as pan-HDAC inhibitors) reduce proteinuria and glomerulosclerosis, thereby increasing survival (41). Epigenetics are implicated in the progression of various kidney diseases, including DN. Histone acetyltransferases’ acetylation activities are balanced by HDACs, which modulate physiological and pathological gene transcription. A class III NAD^+^-dependent HDAC, sirtuin6 (Sirt6), belongs to the sirtuin family, which consists of Sirt1 to Sirt7 that differ in their cellular and tissue distributions. Genes involved in glucose metabolism, DNA repair, telomerase activity, genomic stability, and cellular senescence are regulated by Sirt6-dependent deacetylation of H3K9 or H3K56. Several phenotypes of premature aging have been observed in mice lacking Sirt6. Podocytopathies were associated with a reduced level of Sirt6 in renal biopsies and a reduced glomerular filtration rate (42). In a DN mouse model, Sirt6 deletion exacerbated podocyte injury and proteinuria. Sirt6 suppresses *Notch1* and *Notch4* transcription, negatively regulating Notch signaling. Therefore, Sirt6 has potential as a therapeutic target for proteinuric kidney disease. In addition, many cellular biological processes regulated by Sirt1 are involved in kidney diseases, such as autophagy and apoptosis (43). Enhanced Sirt1 function in podocytes protects diabetic kidneys (44). Hence, histone modifications protect podocytes in hyperglycemia.

### DNA methylation

**Figure 2:**
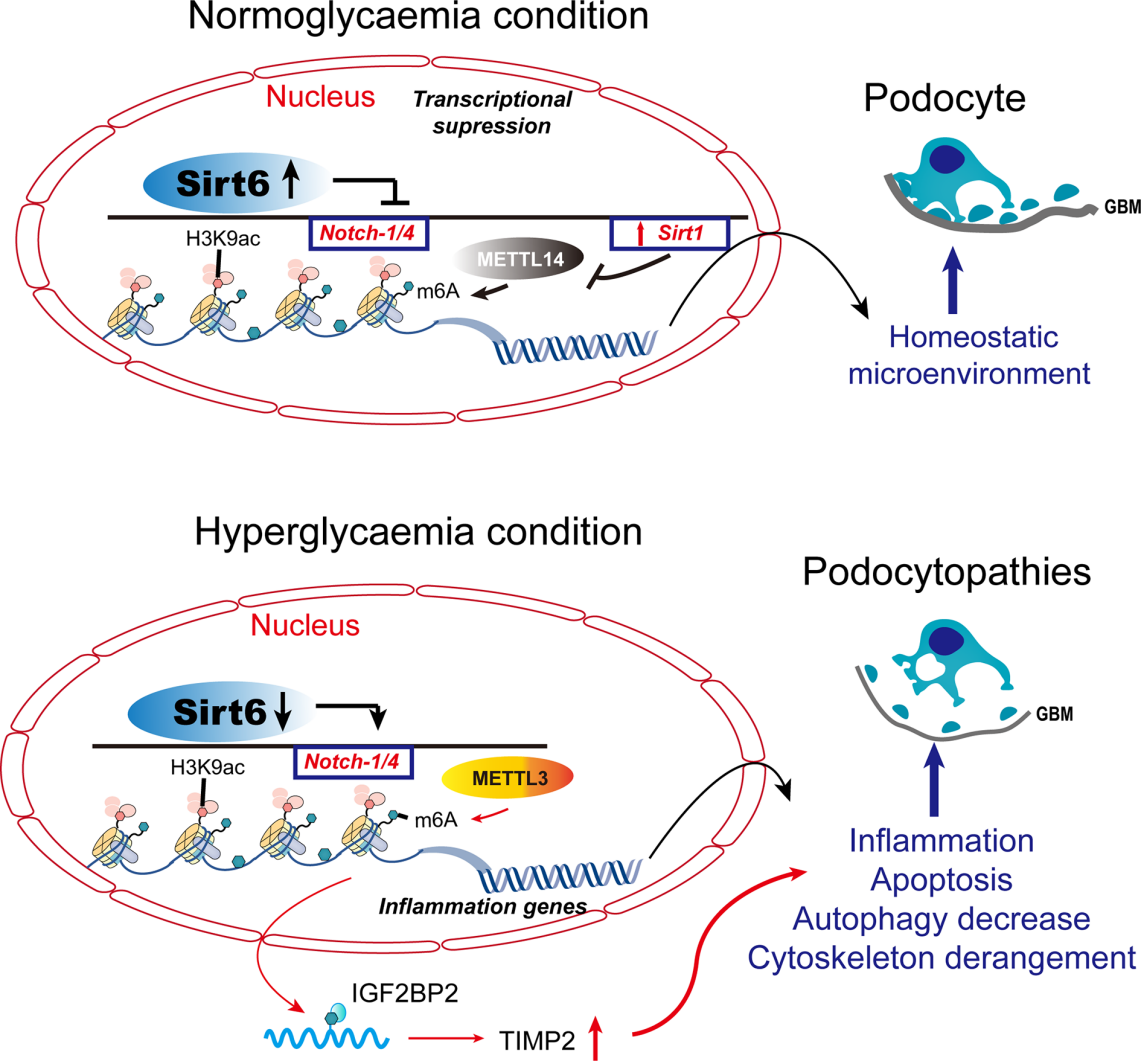
Histone modifications and DNA methylation in podocytopathies. Under normal conditions, Sirt6 inhibits the transcription of *Notch1* and *Notch4* by decreasing H3K9ac levels in the promoter regions of *Notch1* and *Notch4*. METTL14 deficiency inhibits Sirt1 mRNA m6A modification, thereby increasing Sirt1 levels in normal podocytes. METTL3 modulates the m6A modification of TIMP2 in an IGF2BP2-dependent manner and exerts pro-inflammatory and -apoptotic effects. TIMP2, tissue inhibitor of metalloproteinase 2; IGF2BP2, insulin-like growth factor 2 mRNA binding protein 2.

Differences in DNA methylation are associated with predisposition to disease or treatment response. Inter-individual epigenetic differences among individuals can be used as predictive biomarkers of disease susceptibility (45). Thus, DNA methylation levels can be used to distinguish diabetic ESRD and renal complications without diabetes. The METTL3-mediated m6A modification contributes to podocyte injury in DN and targeting m6A by METTL3 has therapeutic potential (46) (**Figure 2**). Sirt1 mRNA was degraded in podocytosis by METTL14-mediated m6A modification. By upregulating Sirt1, podocytes activate autophagy and reduce apoptosis and inflammation, thereby alleviating proteinuria and delaying the progression of podocytopathies. Dysregulation of the RNA m6A modification may be mediated by METTL14 and may be a therapeutic target for podocytopathies (**Figure 2**).

When Sirt6 is reduced under pathological conditions, H3K9ac levels increase in the promoters of *Notch1* and *Notch4*, thereby increasing transcription. Sirt1 mRNA m6A modification and degradation are elevated when podocyte injury induces the expression of METTL14. As a result of Notch-signaling activation, podocyte injury is triggered by inflammation, apoptosis, actin-cytoskeleton degeneration, and autophagy inhibition.

**Figure 3:**
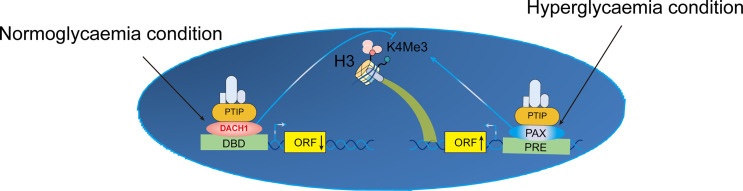
Proposed mechanism of transcriptional activation of K4Me in podocytes. Crosstalk between normal and hyperglycemic conditions in DN. ORF, open reading frame; DBD, DNA-binding domain; H3K4Me3, trimethylation of lysine 4 on histone H3 protein subunit; PRE, Pax response elements; DACH1, Dachshund homolog 1; PTIP, Pax transactivation-domain interacting protein.

Aberrant epigenetic alterations are implicated in the pathogenesis of DKD and modifying the podocyte injury response. DACH1 and Pax transactivation-domain interacting protein (PTIP) target genes are similar. PTIP is recruited by Pax proteins to promoter Pax response elements (PREs), increasing the levels of H3K4Me3 and activating transcription (47). PTIP is also recruited by sequence-specific binding of DACH1 to its target gene promoters, which suppresses transcription and reduces promoter H3K4Me3 levels. When podocyte DACH1 expression is reduced, PTIP is less likely to be recruited to target gene promoters by DACH1. Decreased DACH1 expression in podocytes under hyperglycemic conditions reduces the expression of multiple target genes and downstream signals, thereby damaging podocytes. Downregulation is caused by reduced recruitment of PTIP by the DACH1 target gene promoter, leading to excessive podocyte epigenome activity. Therapeutic interventions aimed at improving podocyte DACH1 activity will correct multiple dysfunctional signals simultaneously. Therefore, testing and development of drugs for DACH1 should be prioritized (**Figure 3**).

### Non-coding RNA

**Figure 4:**
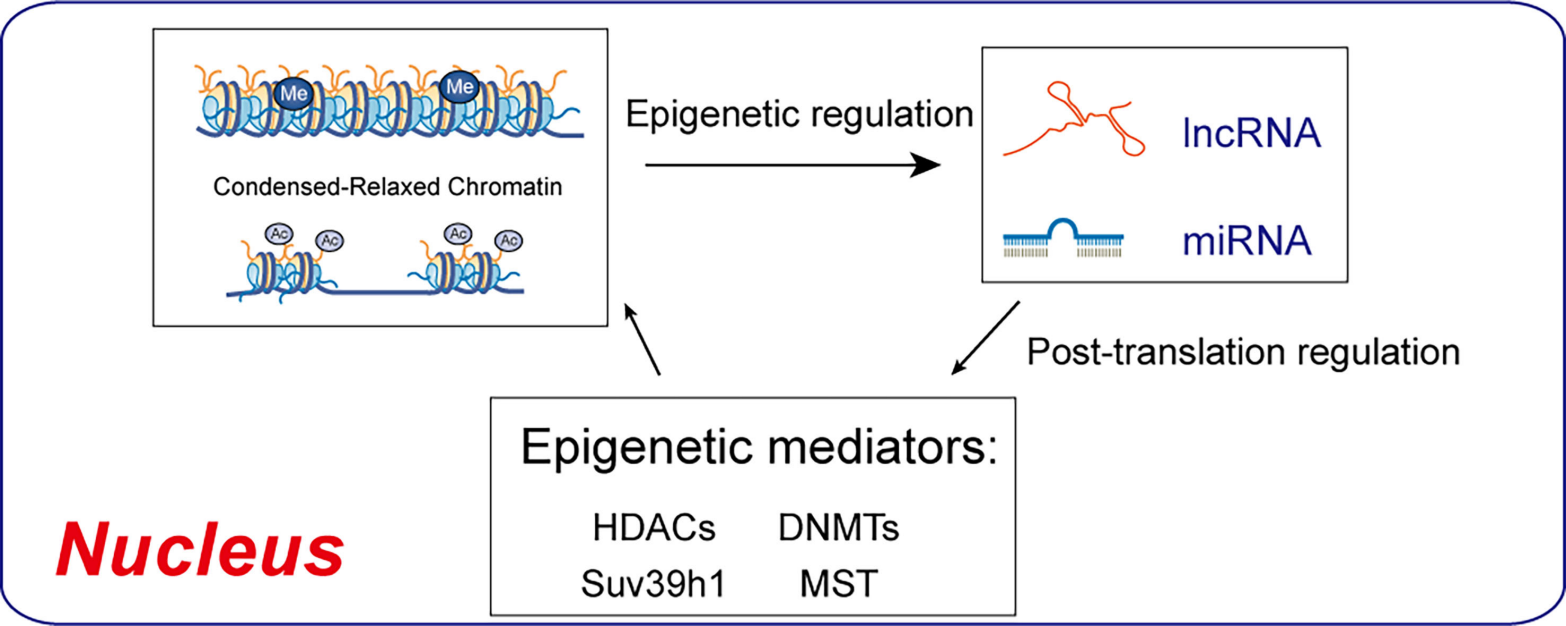
Regulatory interactions between epigenetic machinery and non-coding RNAs. DNMTs and HDACs are epigenetic mediators of DNA methylation and histone acetylation, which result in condensed-relaxed chromatin. lncRNAs and miRNAs modulate epigenetic mediators, thus regulating chromatin remodeling.

Epigenetic determinants such as DNA methylation, histone modification, or RNA can be transferred to the next generation through meiotic products (gametes). DNA methylation and chromatin histone modifications are epigenetic mechanisms, but lncRNAs and microRNAs can also be modified post-transcriptionally (17). MicroRNAs are endogenous non-coding single-stranded RNAs of about 20-24 nucleotides, which can degrade or silence the translation of target gene mRNA by base-pairing with the 3’-UTR sequence (48). MicroRNA regulation of podocyte apoptosis has been a focus of research (48, 49). MicroRNAs regulate autophagy genes, affect the level of autophagy, and are implicated in DN (50, 51). Various miRNA regulatory mechanisms are involved in DN physiological and pathological processes, affecting podocyte apoptosis (49), and are targets for DN prevention and treatment (**Figure 4**).

Long noncoding RNAs (lncRNAs), as competing endogenous RNAs (ceRNAs), confirm the interaction and competitive regulatory mechanisms between lncRNA and microRNA (52, 53, Wang et al., 2022). Enzymes including DNMTs regulate a variety of miRNAs associated with Alzheimer Disease development and progression, including miR-34 a/b/c, miR-107, miR-124, miR-125b, and miR-137 (54). Upregulation of about 40 miRNAs in a lncRNA in the kidneys of diabetic mice promoted DN. lncRNAs have not been systematically studied in podocytopathy to date. miRNAs and epigenetic enzymes play important roles in a variety of diseases.

## ER in podocytopathy during DN progression

Although podocytes play a crucial role in glomerular filtration by forming a slit diaphragm between interdigitating foot processes, the molecular details of protein folding and degradation in the ER are unclear. The ER plays a vital role in maintaining protein synthesis and hemostasis. Two studies have evaluated the pathophysiological roles of the ER in kidney dysfunction (55, 56). ER stress is characterized by the UPR. Sustained ER stress causes cell death, inflammation, and autophagy in the renal tubules. Therefore, inhibiting ER stress may have therapeutic potential for DN (57).

The upregulation of ER stress markers and downstream signaling molecules has been reported in cells, animals, and individuals with DN (55). CHOP, a key regulator of ER stress, was significantly increased in renal biopsies of type 2 DN patients (58, 59). High glucose induces ER stress, podocyte phenotypic switch, and podocyte loss in rat, effects inhibited by exogenous ER molecular chaperones (60, 61). ER stress also triggers podocyte apoptosis. ER markers such as GRP78 are upregulated in diabetic rats’ podocytes (55, 62). Expression of GRP78 and other factors related to ER is increased significantly in HG-induced podocytes, leading to apoptosis (60). A newly hallmark, RTN1A, was found to be increased in the context of ER stress (58). Therefore, ER stress may be a key regulator of podocyte death. Here, based on the three arms of the UPR, we discuss the role of ER stress in inflammation and podocyte apoptosis.

Epigenetic modifications can affect endoplasmic reticulum stress response, resulting in disease risk. MicroRNA expression and DNA and histone methylation patterns are epigenetic phenomena associated with ER stress genes. Some results suggest that the methylation characteristics of leukocyte ER regulatory genes may be related to abdominal/central obesity markers (waist circumference), dyslipidemia and insulin resistance (63). However, the finding provides insight into the relationship between disorders and epigenetics as well as complications resulting from endoplasmic reticulum stress, further research is needed to confirm this.

## Signaling pathways involved in inflammation and apoptosis

*TLR4* knockout mice showed reduced albuminuria, renal dysfunction, inflammation, and fibrosis after STZ treatment. Diabetes-induced podocyte loss was reduced by deletion of Tlr4 despite tubular injury suppression (64, 65). High glucose upregulated *TLR4* expression in cultured podocytes by triggering ROS production and NF-κB activation (66). By activating the protein kinase C pathway and NADPH oxidase (NOX), high glucose increases TLR2 and TLR4 expression in monocytes (67). High glucose-induced upregulation of IL-6 and CCL2 was inhibited by silencing *TLR4*, while TLR2-deficient tubular cells showed no significant reduction in cytokine production. High glucose induced the expression of *TLR4* but not of *TLR2* in mesangial cells (68). GIT27, an anti-TLR agent, showed an anti-proteinuric effect in db/db mice treated with an immunomodulator that targets macrophages by blocking TLR2, TLR4, and TLR6 signals (69). Additionally, GIT27 inhibited cytokine production induced by glucose and free fatty acids in cultured podocytes (11). Therefore, TLR4 signaling is implicated in inflammation in hyperglycemic patients.

Akin to TLRs, NLRs play an important role in innate immunity by triggering pro-inflammatory cascades when pathogen-associated molecular patterns (PAMPs) and damage-associated molecular patterns (DAMPs) trigger innate immunity (70, 71). A variety of human diseases are caused by the NLRP3 inflammasome complex, including cancer, liver disease, and DKD. NLRP3 inflammasome activation is a two-step process. First, priming by PAMP- or DAMP-induced activation of TLR signaling, resulting in NF-κB-dependent expression of NLRP3, pro-IL-1β, and pro-IL-18. Second, various cellular mechanisms, such as potassium efflux, pore-forming toxins, calcium influx, mitochondrial dysfunction, and intracellular ROS production, trigger the formation of the NLRP3 complex and activation of CASP1 (70). Nod-like receptor protein 3 (NLRP3) inflammasome activation is responsible for the accumulation of intracellular or plasma S-adenosyl-homocysteine, promoting hyperglycemia-induced podocyte injury (72). Nephropathy in db/db mice was preceded by inflammasome activation in podocytes and endothelial cells (73). Diabetes-induced hyperglycemia increased thioredoxin-interacting protein (TXNIP) expression in the kidneys of diabetic mice, resulting in NOX and inflammasome activation (3, 74), podocyte loss and the onset of albuminuria (75). In mice, angiotensin II also activated NLRP3 inflammasomes, produced mitochondrial dysfunction, and led to nephron and podocin loss, as well as albuminuria (76). Therefore, NLRP3 inflammasome activation induces podocyte apoptosis and mitochondrial dysfunction, resulting in proteinuria. Additionally, *NLRP3-*knockout mice with STZ-induced DKD had improved renal function, glomerulosclerosis, tubulointerstitial inflammation, and renal fibrosis (77).

**Figure 5:**
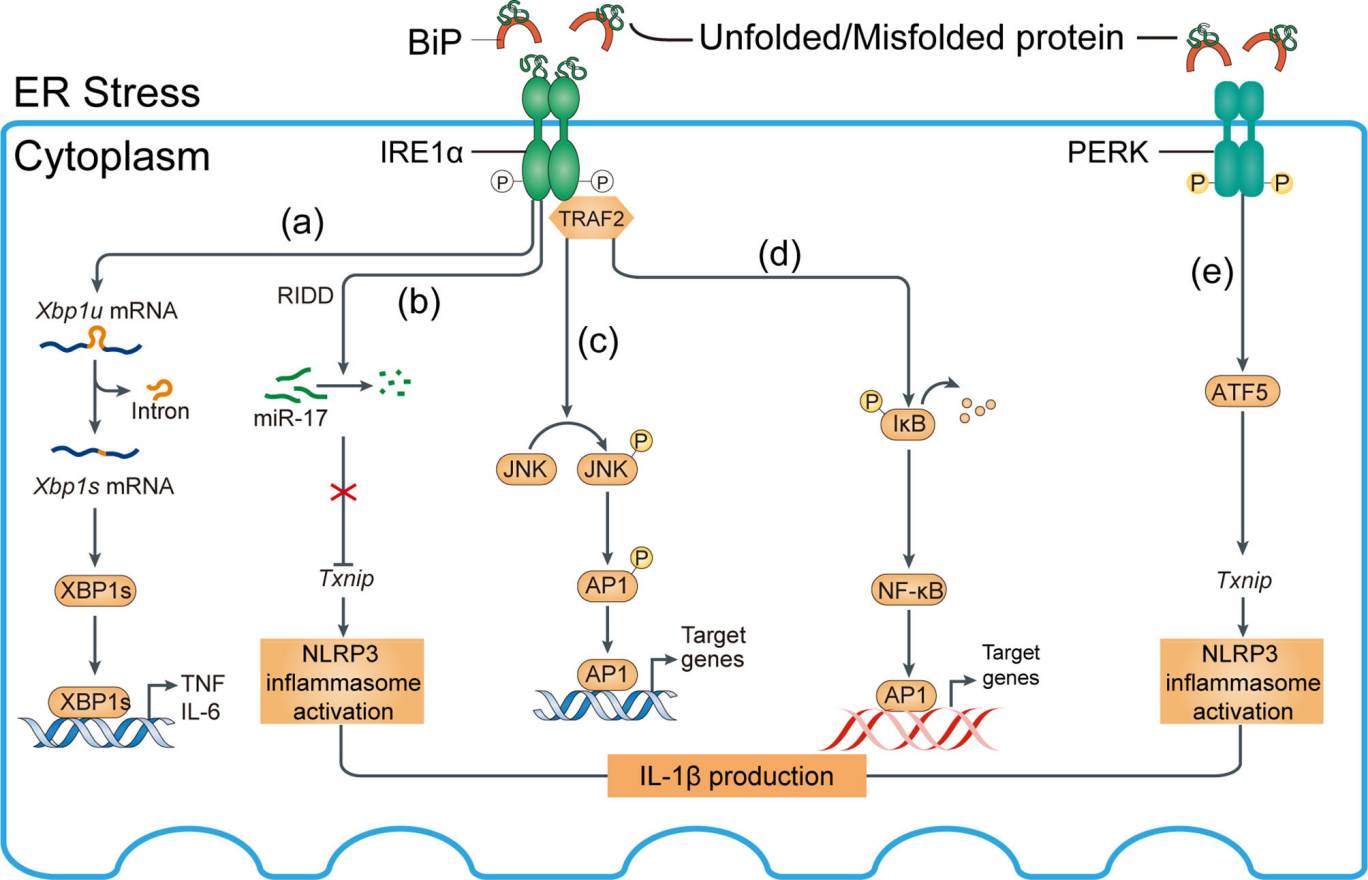
Mechanisms of ER stress-induced inflammation. Inositol-requiring enzyme 1α (IRE1α) and PKR-like ER kinase (PERK) activation induce inflammasome activation. **(A)** IRE1α activation and subsequent splicing of X-box binding protein 1 (Xbp1) produces the transcription factor XBP1s, which directly binds the promoters of tumor necrosis factor (TNF) and interleukin-6 (IL-6). **(B)** Regulated IRE1α-dependent decay (RIDD)-dependent degradation of miR-17, which in unstressed conditions represses thioredoxin-interacting protein (Txnip), increases the *Txnip* level, NLRP3 inflammasome activation, and IL-1β expression. **(C)** Activated IRE1α forms a complex with TNF receptor-associated factor 2 (TRAF2) to induce phosphorylation of Jun N-terminal kinase (JNK) and upregulation of proinflammatory genes *via* activated activator protein 1 (AP1). **(D)** IRE1α–TRAF2 complex recruits IκB kinase (IKK); subsequent phosphorylation and degradation of IκB releases nuclear factor-κB (NF-κB) for nuclear translocation. BiP, binding immunoglobulin protein. **(E)** *Txnip* can be induced by the PKR-like ER kinase (PERK)-activating transcription factor 5 (ATF5) pathway to induce inflammasome activation.

**Figure 6:**
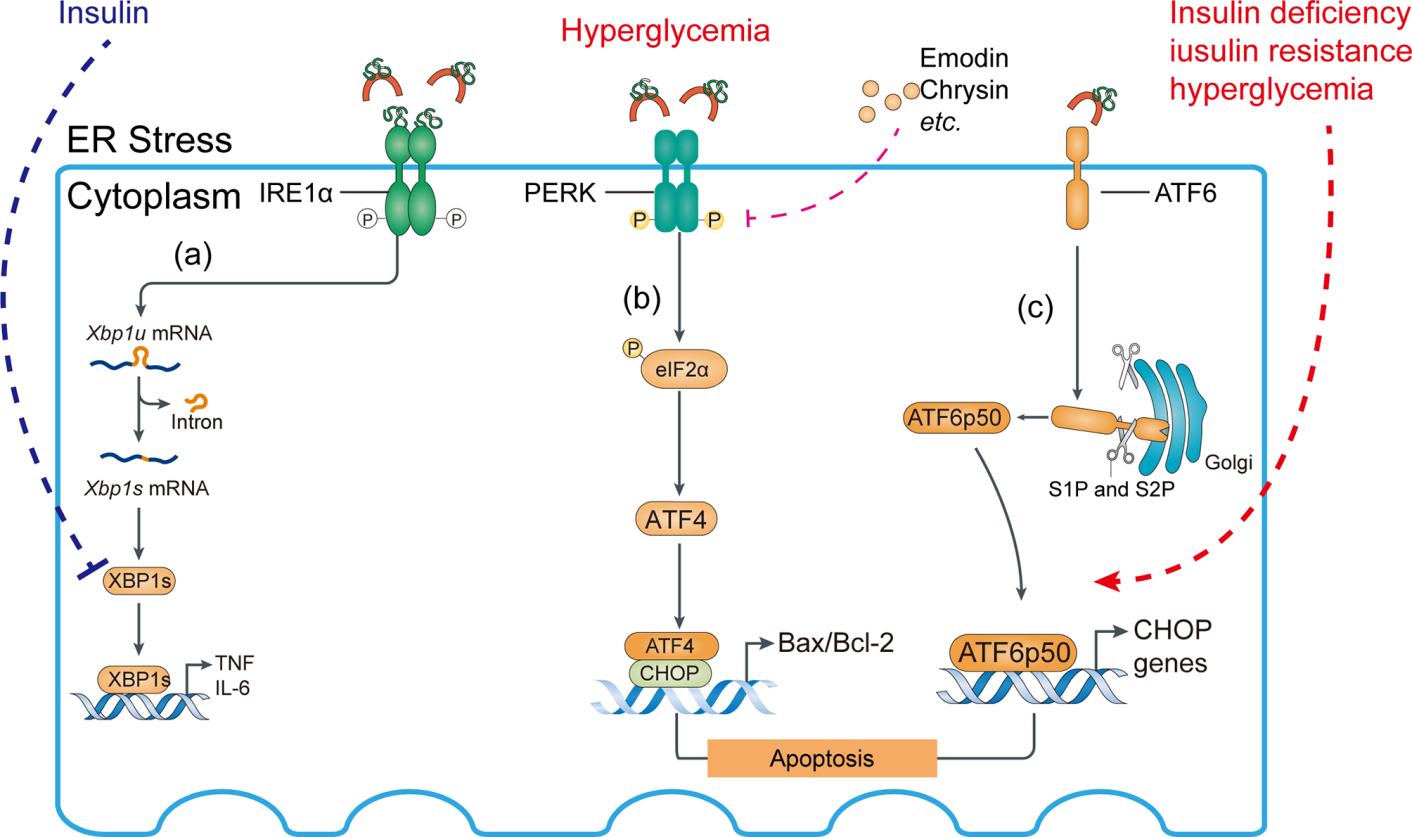
Activation of ATF6 pathway and abnormal IRE1 pathway may aggravate ER stress response. **(A)** Role of the IRE1 pathway in the ER stress response. Insulin signaling inhibits the interaction of XBP1s with the phosphatidylinositol 3-kinase (PI3K) regulatory subunits p-85α and p-85β, suppressing proinflammatory cytokine production. **(B)** Compounds such as emodin and chrysin inhibit PERK activation and decrease the Bax/Bcl-2 ratio in hyperglycemia, preventing podocyte apoptosis. **(C)** Insulin deficiency, insulin resistance, and hyperglycemia aggravate site 1 protease (S1P)- and S2P-mediated cleavage of ATF6α, allowing their fragments to translocate to the nucleus.

The ER chaperone immunoglobulin-binding protein (BiP) or glucose-regulated protein 78 (GRP78) mediates the UPR signaling network. The UPR consists of three major branches, which are initiated by the activation of three protein sensors—inositol-requiring enzyme 1α (IRE1α), activating transcription factor 6 (ATF6), and protein kinase RNA (PKR)-like ER kinase (78, 79) (**Figures 5**, **6**). Under physiological conditions, the transducers are bound to BiP and are inactive. Accumulation of misfolded or unfolded proteins under stress detaches BiP from the transducers, simultaneously activating them (55). Subsequently, spliced Xbp1 (XBP1s), which encodes a potent transcriptional activator, is cleaved by IRE1-mediated sequence-specific cleavage (**Figure 5A**). Moreover, ER stress reduces insulin signaling during the development of diabetes *via* activation of JNK, plus it induces pancreatic apoptosis, which worsens diabetes complications (80). Insulin supplementation can inhibit the transcriptional regulation of XBP1s and thereby alleviate inflammation (**Figure 6A**).

After separating from BiP, UP-mediated phosphorylation of protein kinase RNA-like ER kinase (PERK) activates downstream activating transcript factor 5 (ATF5) (81–83) and its target genes such as *Txnip*, thus inducing inflammation of kidney cells, such as podocytes (84) (**Figure 5E**). PERK is also implicated in macrophage immunosuppression and its downstream targets include ATF4, ATF5, and TXNIP (85).

Eukaryotic translation initiation factor-2α (eIF2α) phosphorylation reduces the translation of most mRNAs by inhibiting the delivery of the initiator Met tRNAi to the initiation complex, leading to reduced protein translation and protein loading into the ER, thereby alleviating the early stage of ER stress (86–88). Acute diseases typically benefit from an adaptive UPR, but chronic diseases (such as hyperglycemia) may be adversely affected by long-term or continuous UPR activation (89). In fact, eIF2α promotes the translation of a number of mRNAs containing short upstream open reading frames such as ATF4, activating downstream factors (*e*.*g*., *CHOP*, Bax/Bcl-2 transcriptional expression) and inducing apoptosis (86). Unfortunately, the mechanism responsible for the transition of the UPR from an adaptive to a cytotoxic response is unclear (**Figure 6B**).

In the Golgi apparatus, proteins are continuously cleaved to release cytosolic effectors or proteins from the membrane and allow them to enter the nucleus. Under ER stress, the transport of ATF6 from the ER to the Golgi apparatus is mediated by Golgi-resident site 1 protease (S1P) and S2P in an intramembrane region to release the cytoplasmic DNA binding protein portion, the ATF6 fragment. The ATF6 fragment translocates to the nucleus to induce gene expression (90), including UPR target genes (**Figure 6C**).

All branches of the UPR may be linked to podocyte apoptosis. The UPR is activated by increased expression of BiP, p-IRE1α, p-eIF2α, CHOP, ATF-6, and XBP1s in hyperglycemic podocytes *in vitro* and *in vivo* (91). ER stress activates the PERK pathway, upregulating the expression of downstream factors related to apoptosis such as Bax, leading to podocyte apoptosis. Increased phosphorylated PERK (p-PERK), phosphorylated eIF2α (p-eIF2α), ATF4 and CHOP increased the Bax/Bcl-2 ratio, promoting podocyte apoptosis (92, 93). By contrast, there are reversed results after using shRNA or knockdown PERK directly and thus podocyte apoptosis was controlled. Inhibition of PERK maintained the structure and function of the glomerular filtration barrier by increasing the production of the slit-diaphragm proteins podocin/nephrin (92). However, there is no information on the other two UPR branches. Confusingly, suppression of the PERK pathway under ER stress exacerbated β-cell apoptosis in thapsigargin-induced primary human islets, mouse islets, and INS-1 beta cells (94). It is possible that activated PERK induces phosphorylation of eIF2α, suppressing protein translation and loading into the ER and thereby alleviating ER stress. Therefore, PERK is implicated in podocyte apoptosis in DN, but the mechanism warrants further research.

## Targets for podocytopathy therapy

### Epigenetic mechanisms

Histone modification and DNA methylation status are altered by hyperglycemia, causing chromatin structure to change from concentration (K9Me and K27Me) to relaxation (K4Me), activating the expression of pathogenic genes (**Figure 4**) (95). Various mechanisms can enable transcription factors and cofactors to access promoters and enhancers through histone acetylation, to enhance gene expression (96). However, insulin or anti-diabetic drugs continue to promote the expression of pathogenic genes after normoglycemia. The use of epigenetic drugs or induction of site-specific changes, such as locus modification or DNA methylation, may eliminate podocytopathies.

The structure-activity relationship (SAR) approach based on the resveratrol structure showed BF175 to be an effective SIRT1 agonist, activated by the downstream transcription factor PGC-1α. The protective effect of BF175 on diabetes podocytes was SIRT1 dependent (44). METTL14 expression was highest in mice with Adriamycin-induced DNA and an elevated m6A RNA level. Furthermore, METTL14 levels were increased in renal biopsies from diabetics, models of DN, and human podocytes cultured with advanced glycation end products. Mice with podocyte-specific METTL14 deletions had improved glomerular function and alleviated podocyte injury, which was characterized by activation of autophagy and inhibition of apoptosis and inflammation (97).

There is an increase in METTL3 in podocytes from renal biopsy samples from patients with DN, which is associated with renal damage, in addition to METTL14 (46). *METTL3 Flox/Flox (METTL3^fl/fl^)* mouse lines have been used as *METTL3-*deficient models (97). Further, STZ-induced diabetic mice with METTL3 podocyte-conditional knockouts showed significant improvement of podocyte injury and albuminuria. As a result of METTL3, TAB3 was modified by m6A, and its stability increased in a manner dependent on IGF2BP2. Genetic and pharmacological inhibition of METTL3 can attenuate renal injury and inflammation, suggesting that the METTL3/TAB3 axis is a potential therapeutic target in podocytopathies (98).

TXNIP-mediated oxidative stress and NLRP3 inflammasome activation has been decribed previously. A study by Yunjun Xiao et al. has shown that H3K27me3 is reduced trimethylated and its occupancy at promoters of early growth response 1 (EGR1) is enhanced by histone methyltransferase enhancer of zeste homolog 2 (EZH2). S-adenosylhomocysteine (SAH) is hydrolyzed by SAH hydrolase (SAHH) to homocysteine and adenosine. Adenosine dialdehyde (ADA), an inhibitor of SAHH, accumulates intracellular or plasma levels of the SAH levels and may exert as a regulator to hyperglycaemia-induced podocytopathy (72).

KLF4 epigenetics regulate podocyte phenotype and function, and the podocyte epigenome can be used to ameliorate proteinuria. A ChIP analysis of DNA methyltransferases (DNMTs) showed that DNMT1 binding to the nephrin promoter region was significantly reduced in KLF4-overexpressing podocytes, but its binding to the vimentin promoter was unaffected (99). To confirm that the transient upregulation of KLF4 has an extended effect on podocyte function, the *Tet-On* gene induction system induced by doxycycline was used to construct mice with KLF4-inducible and podocyte-specific overexpression. Doxycycline increased the expression of KLF4, and KLF4 was co-located with the podocyte marker nephrin, but not with the endothelial or mesangial marker desmin. Therefore, doxycycline may play an important role in the epigenetic regulation of KLF4 in podocytes.

Hyperglycemia suppresses RCAN1 expression in cultured podocytes, which is alleviated by pretreatment with the DNA methyltransferase inhibitor 5-Aza-2’-deoxycytidine. Mechanistically, increased expression of RCAN1 decreased hyperglycemia-induced nuclear factor of activated T cells (NFAT) transcriptional activity. Increased RCAN1 expression also stabilized actin cytoskeleton organization, consistent with inhibition of the calcineurin pathway (100). Hence, 5-Aza-20-deoxycytidine may suppress the expression of CXCL2, NFAT, and Wnt6 in hyperglycemic podocytes (**Table 1**).

**Table 1:** Reports of epigenetic drug treatment on podocytes during DN progression.

No.	Agents	Target genes/proteins	Signaling Route	Trends	Experimental model	Cytokines	Epigenetic machinery in podocytes
1	BF175	Sirt1	Sirt1	↑	Diabetic OVE26 mice	(NA)	Improving the mitochondrial function and homeostasis
			PGC-1α	↓			
2	Adriamycin	METTL14	Sirt1	↓	C57BL/6J mice	Inhibited MCP-1, IL-6 and TNF-α	Promoting autophagy and inhibiting apoptosis and inflammation
3	Anti-METTL3 antibody	METTL3	p-p65 NF-κB	↓	Mouse podocytes (MPC5)	Inhibited TNF-α, interleukin-1β (IL-1b), and MCP-1	Relaxed chromatin and increased cell content
4	Cpd-564	METTL3	NF-κB	↓	*METTL3* cKO mice	(NA)	Inhibiting the inflammatory response and programmed cell death
5	Adenosine dialdehyde (ADA)	S-adenosylhomocysteine hydrolase (SAHH) and H3K27me3 at EGR1 promoter	TXNIP signaling	↑	C57BL/6J mice and human podocyte cell line	NLRP3 inflammasome activation	Inhibiting S-adenosylhomocysteine but aggravated oxidative stress
6	Doxycycline	DNMT1/H3K9ac	KLF4 expression	↑	human podocyte cell line	Increased nephrin	Increases nephrin promoter activity
7	5-Aza-2’-deoxycytidine	*RCAN1*	Calcineurin-NFAT	↓	*RCAN1* ^-/-^ mice and human podocyte cell line	Decreased CXCL2	Reduces podocyte apoptosis and stabilized actin cytoskeleton organization

↑ Indicate increased expression, ↓ Indicate decrease.

NFAT, nuclear factor of activated T cells; RCAN1, regulator of calcineurin 1.

**Figure 7:**
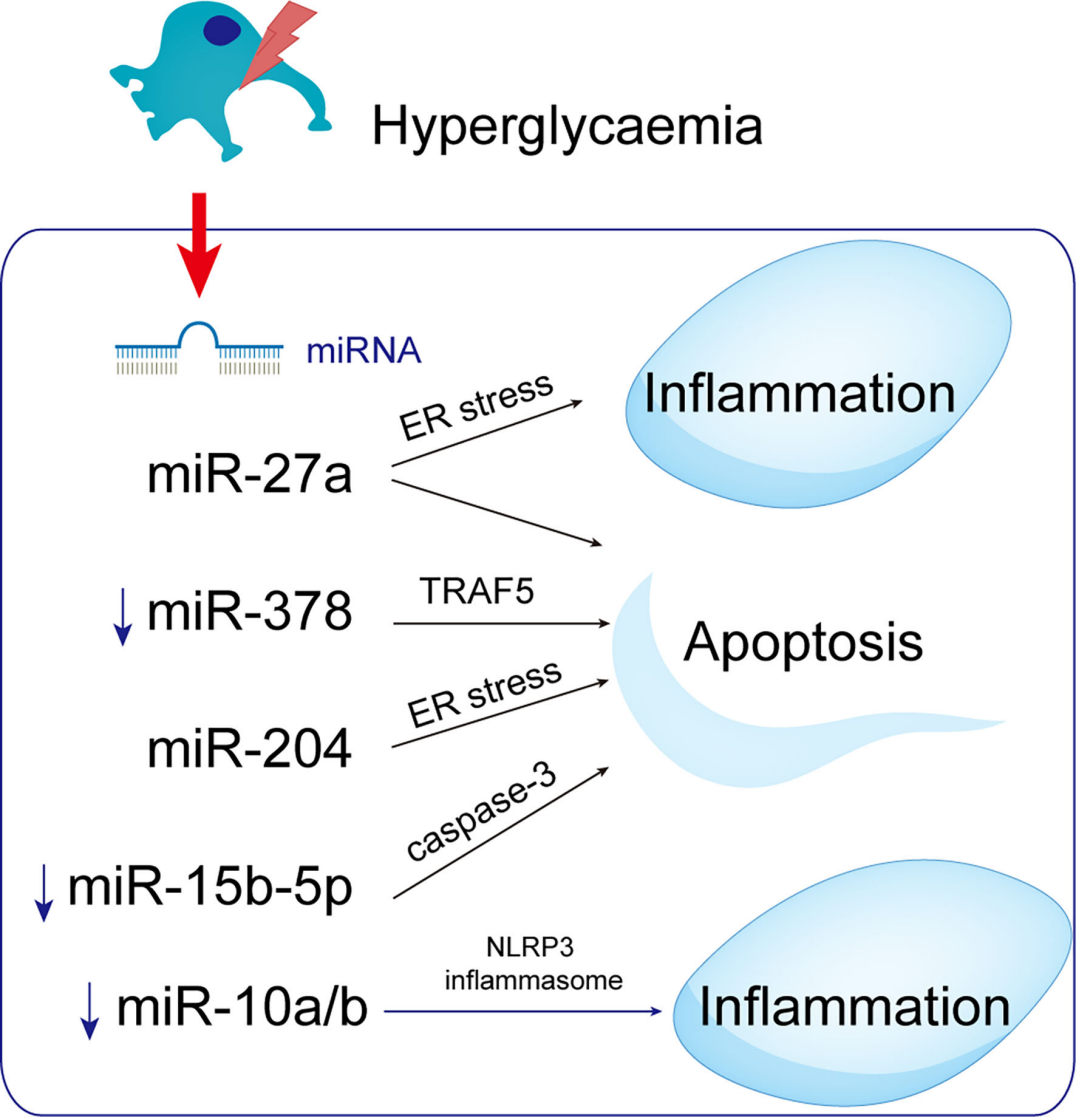
MicroRNAs with potential roles in podocytopathies. Hyperglycemia up- or down-regulates miRNAs in podocytes, resulting in podocytopathies. ↑ Indicate increased expression, ↓ Indicate decrease.

miRNA-mediated epigenetic regulation of inflammatory gene expression is implicated in diabetes complications (101). MicroRNA-10 targets the NLRP3 inflammasome to suppress inflammation in diabetic kidneys. Patients with DKD and diabetic mice with miR-10a/b had low levels of miR-10a and -10b, which are expressed predominantly in podocytes. When miR-10a and b were delivered to the kidneys, NLRP3 inflammasomes were not activated and renal inflammation was prevented. By contrast, miR-10a/b knockdown increased activation of the NLRP3 inflammasome. Thus, upregulation of miR-10a/b could prevent podocytopathy in hyperglycemics (102) (**Figure 7**).

miRNA-27a is upregulated in podocytes of DN patients, possibly enhancing podocyte migration, diffusion, and apoptosis (103). Furthermore, miRNA-27a causes podocyte injury and apoptosis by targeting ER stress (52). The expression of miR-378 was downregulated in DN rats and high glucose-mediated podocytes, promoting podocyte apoptosis, which was reversed by inhibiting the expression of tumor-necrosis factor receptor associated factor 5 (TRAF5) by miR-378 (104). Moreover, miRNAs may be related to DN pathogenesis because they affect the expression of genes in the UPR signaling pathways. MiR-204 directly inhibits its target PERK under ER stress, exacerbating ER stress-induced apoptosis (105). MiR-204 targets the insulin transcription factor MAFA and thereby inhibits insulin translation and synthesis, indirectly activating ER stress (105–107). MiR-15b-5p was downregulated in patients with DN and could cause increased apoptosis in human kidney cells supported by elevated active caspase-3 and decreased viability and proliferation (108), linking miRNAs to the pathogenesis of DN.

As a competitive RNA (ceRNA), lncRNAs interact with and competitively regulate microRNAs (52, 53, 109). When lncRNA MALAT1 is decreased, microRNA let-7f is upregulated and KLF5 is inhibited, reducing podocyte damage in DN (110). miR-130a-3p competes with TLR4 as an endogenous sponge, as well as TLR4 as a miR-130a-3p target gene. As a result of miR-130a-3p/TLR4 crosstalk, MIAT knockdown protected podocytes from hyperglycemia-induced damage. Reduced MIAT expression restores slit-diaphragm integrity, attenuates foot process effacement, prevents dedifferentiation, and suppresses mitogenic catastrophe in podocytes during hyperglycemia. In response to ER stress, lnc-MGC and miR-379 cluster miRNAs are increased, and DN phenotypes (hypertrophy and fibrosis) are aggravated (111). Those findings show that miRNAs and lncRNAs are related to ER stress.

The lncRNA plasmacytoma variant translocation 1 (PVT1; 1.9 kb) is linked to kidney disease and encodes many alternative transcripts but not a protein. Silencing of PVT1 inhibits podocyte damage and apoptosis *via* the forkhead box A1 (FOXA1) in DN, which is of clinical importance (112). On human chromosome 20, small nucleolar RNA host gene 17 (SNHG17) is highly expressed in colorectal cancer tissues (113). SNHG17 controls mitophagy-induced apoptosis in diabetic podocytes. lncRNA SNHG17 knockdown promotes Parkin-dependent mitophagy and reduces apoptosis of podocytes by regulating the degradation of mammalian sterile 20-like kinase 1 (MST1) (114).

**Figure 8:**
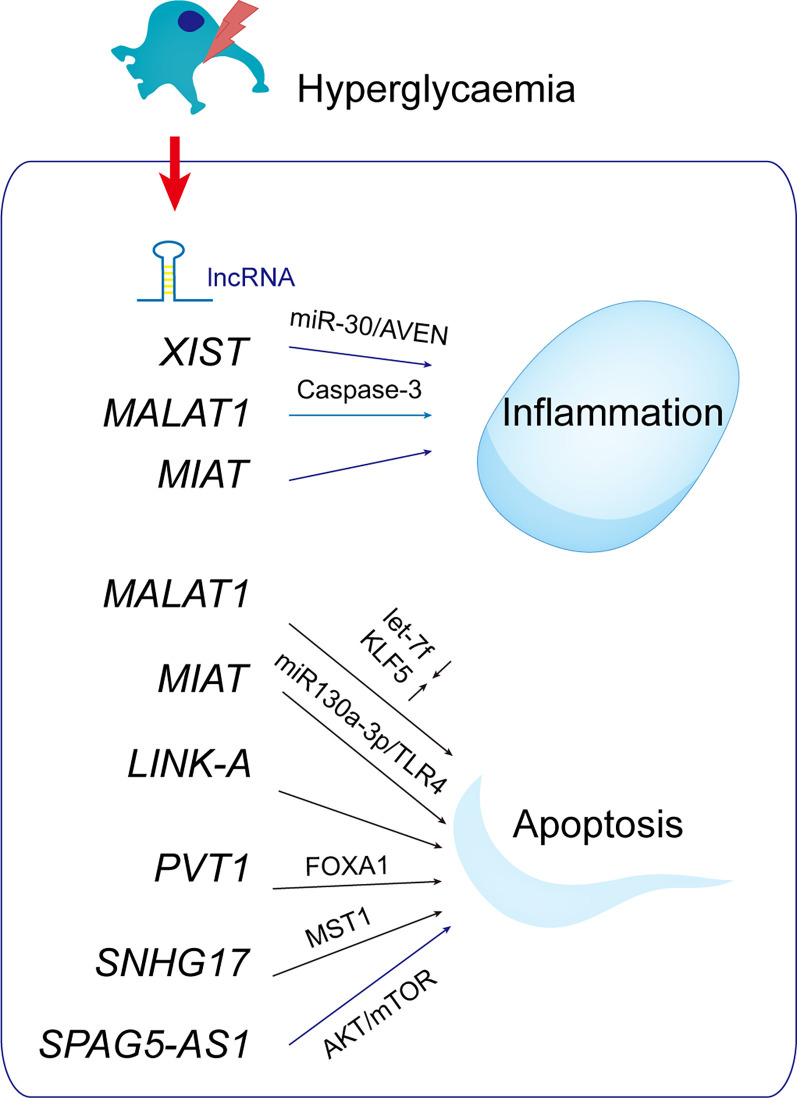
LncRNAs with potential roles in podocytopathies. Hyperglycemia up- or down-regulates lncRNAs in podocytes, resulting in podocytopathies.

The AKT/mTOR pathway inhibits autophagy in a variety of cell types, including cancer cells, cardiomyocytes, and podocytes (115). The SPAG5/AKT/mTOR pathway inhibits podocyte autophagy and aggravates apoptosis. Therefore, SPAG5-AS1/SPAG5 has therapeutic potential for podocytopathies and DN (116) (**Figure 8**).

### Therapies targeting in ER stress

There has been considerable interest in targeting ER stress as a therapy to improve DN. A variety of natural compounds inhibit ER stress and so may have therapeutic value for podocytopathies.

Natural products that modulate ER stress are listed in **Table 2**. ER stress can be regulated by inhibiting UPR sensors and their downstream factors. Emodin (93), chrysin (92), berberine (119), huaiqihuang (118) and epigallocatechin 3-gallate (EGCG) (122) inhibit the PERK signaling pathway to alleviate ER stress and improve DN. MC-TG (117) reduces ER stress and inflammation, possibly by inhibiting IRE1/NF-κB. Also, OA and NAC (120) inhibit the three UPR sensors to suppress ER stress. ER stress is triggered by impaired SERCA2 activity or expression, hampering Ca^2+^ homeostasis (124). AS-IV may alleviate ER stress by increasing SERCA2 expression, thus restoring intracellular Ca^2+^ homeostasis (121). ER chaperones such as 4-phenylbutyrate (4-PBA) and ursodeoxycholic acid (UDCA) enhance the folding capacity of the ER (91, 123). Therefore, natural compounds have potential for inhibiting ER stress and thus attenuating DN.

**Table 2:** Therapies targeting ER stress.

Agents	Mechanisms
Emodin^93^	Inhibition of PERK
Chrysin^92^	
MC-TG^117^	Inhibition of IRE1α/NF-kB
Huaiqihuang^118^	Inhibition of GRP78, CHOP
Berberine^119^	
OA or NAC^120^	Suppression of ER stress
AS-IV^121^	Upregulation of SERCA
EGCG^122^	Inhibition of p-PERK, GRP78, CHOP
4-PBA and UDCA^60^^;^ 123	Enhances protein folding efficiency

ER: endoplasmic reticulum; 4-PBA, 4-phenylbutyric acid; IRE1α, inositol-requiring enzyme 1α; PERK, PRKR-like ER kinase; UDCA, ursodeoxycholic acid; XBP1, X box-binding protein 1; NF-κB, nuclear factor-κB; GRP78, glucose-regulated protein 78; CHOP, C/EBP homologous protein; EGCG, Epigallocatechin-3-gallate; OA, Oleanolic acid; NAC, N-acetylcysteine; MC-TG, Terpene glycoside component from Moutan Cortex; AS-IV, Astragaloside IV; Sarco/endoplasmic reticulum Ca^2+^-ATPase2 (SERCA2 ) SERCA 2.

Preventing ER stress can improve DN and attenuate oxidative stress. The effect of hyperglycemia-mediated oxidative stress on podocytes suggests that podocyte oxidation is activated by upregulating ER markers after exposure to high glucose for 24 hours, whereas inhibition of ER stress by ER inhibitors diminishes oxidative stress and exerts a renoprotective effect (60). Similarly, crosstalk between ER stress and oxidative stress mediated by aldosterone contributes to podocyte injury, which can be ameliorated by berberine (119). Therefore, novel therapeutic strategies aimed at attenuating mitochondrial dysfunction may ameliorate palmitic acid-induced podocyte injury.

Autophagy delivers proteins and damaged organelles to lysosomes for degradation and recycling to maintain intercellular hemostasis. Autophagic self-repair is important in neurons, podocytes, and other cells in the anaphase of division because their differentiation and proliferation are limited (125). Resveratrol attenuated hyperglycemia-induced apoptosis by activating autophagy in db/db mice and podocytes (51). miR-383-5p overexpression significantly inhibited autophagy and enhanced apoptosis, effects reversed by resveratrol. Therefore, modulation of autophagy may be a novel therapeutic approach for DN. Activated eIF2α upregulates the mRNA level of ATF4, which transcriptionally regulates autophagy factors to induce autophagy. Also, phosphorylation-dependent selective translation upregulates Atg12 expression and stimulates Atg5-Atg12-Atg16 complex formation, thereby initiating autophagy. This could promote cell survival *via* autophagic decomposition of cellular components to generate energy under adverse conditions (126). Therefore, modulation of the crosstalk between ER stress and autophagy may have therapeutic potential for DN.

## Conclusion

A hyperglycemic environment can induce podocytopathy. As podocyte function declines, so does glomerular function. Podocyte inflammation and apoptosis are the main causes and characteristics of early proteinuria in DN. Although epigenetic mediators (*e*.*g*., HDACs and DNMTs) are not genes, they regulate inflammation and apoptosis, and modulate the inflammatory response and apoptosis induced by ER stress in hyperglycemia. Therefore, protecting podocytes from deleterious effects can maintain the filter membrane, and thereby prevent DN. Natural drugs may also be useful for treating podocytopathy by transcriptionally regulating downstream factors of ER stress. We reviewed the epigenetic mechanisms and natural substances that regulate ER stress and inflammatory signaling pathways in podocytosis, with the aim of identifying new therapeutic targets in progressive DN.

## Author contributions

XW and JR reviewed the literature and drafted the manuscript. JZ and YL participated in the conception and interpretation of the relevant literature for the manuscript. The final version of this review has been edited, revised critically, and approved by all authors.
